# Antibodies Processed Using High Dilution Technology Distantly Change Structural Properties of IFNγ Aqueous Solution

**DOI:** 10.3390/pharmaceutics13111864

**Published:** 2021-11-04

**Authors:** Nikita Penkov

**Affiliations:** Laboratory of Optical and Spectral Analysis Methods, Institute of Cell Biophysics RAS, Federal Research Center “Pushchino Scientific Center for Biological Research of the Russian Academy of Sciences”, 142290 Pushchino, Russia; nvpenkov@rambler.ru

**Keywords:** terahertz, new properties, lactose, interferon-gamma, high dilutions, structural properties, water hydrogen bonds

## Abstract

Terahertz spectroscopy allows for the analysis of vibrations corresponding to the large-scale structural movements and collective dynamics of hydrogen-bonded water molecules. Previously, differences had been detected in the emission spectra of interferon-gamma (IFNγ) solutions surrounded by extremely diluted solutions of either IFNγ or antibodies to IFNγ without direct contact compared to a control. Here we aimed to analyse the structural properties of water in a sample of an aqueous solution of IFNγ via terahertz time-domain spectroscopy (THz-TDS). Tubes with the IFNγ solution were immersed in fluidised lactose saturated with test samples (dilutions of antibodies to IFNγ or control) and incubated at 37 °C for 1, 1.5–2, 2.5–3, or 3.5–4 h. Fluidised lactose was chosen since it is an excipient in the manufacture of drugs based on diluted antibodies to IFNγ. After incubation, spectra were recorded within a wavenumber range of 10 to 110 cm^−1^ with a resolution of 4 cm^−1^. Lactose saturated with dilutions of antibodies to IFNγ (incubated for more than 2.5 h) changed the structural properties of an IFNγ aqueous solution without direct contact compared to the control. Terahertz spectra revealed stronger intermolecular hydrogen bonds and an increase in the relaxation time of free and weakly bound water molecules. The methodology developed on the basis of THz-TDS could potentially be applied to quality control of pharmaceuticals based on extremely diluted antibodies.

## 1. Introduction

Modern research into the physics of water and aqueous solutions shows that the activity of extremely diluted aqueous solutions [1] is determined by the preparation process, which combines an external treatment (electromagnetic field exposure or mechanical vibration) and multiple sequential dilutions of the original substance. The external treatment of water is, in its essence, a complex physical process leading to changes in the physicochemical properties of the solvent [2]. Accordingly, it has been shown that exposure to 36 GHz or 42 GHz microwaves modifies the physical and biological properties of water. Moreover, this effect will persist for tens of minutes or even longer after the electromagnetic field generator is turned off [3].

A recent finding is important in understanding the mechanisms of activity for extremely diluted solutions and how they are influenced by physical factors. It has been discovered that the preparation of effective extremely diluted solutions is impossible under hypomagnetic conditions [4]. Many works have been devoted to identifying various nanoscale objects in such dilutions [1,2,5,6,7]. These studies have made it possible to substantiate the structural features of extremely diluted solutions (their functional activity aside). The physical mechanism of this activity may lie in the ability of aqueous solutions subjected to a physical treatment to emit waves in the submillimeter frequency range. It has been demonstrated that water, after its exposure to a femtosecond laser, emits radiation in the terahertz frequency range [8,9].

A number of publications have recently appeared on the biological activity of extremely diluted solutions of antibodies to IFNγ [10,11,12,13]. It was shown that this activity is based on the ability of extremely diluted solutions of antibodies to modify the conformation of their target molecule, IFNγ. Nuclear magnetic resonance (NMR) analysis demonstrated that adding extremely diluted solutions of antibodies to an IFNγ aqueous solution resulted in conformational changes in the IFNγ molecule with an accompanied modification of its biological activity [14,15]. In one recent study [16], a fundamentally new ability of the extremely diluted solutions’ mechanism of action was discovered. It would seem that their effect can be realised without direct contact with the substance (i.e., distantly). We detected specific changes in the mid-IR region emission spectrum after an aqueous solution of IFNγ was placed in a sealed plastic tube and immersed in extremely diluted solutions of either IFNγ or antibodies to IFNγ for 1 h. The effect of the high dilution technique used in preparing the test samples on the structural properties of the IFNγ solution is one possible explanation.

Terahertz spectroscopy has been developing rapidly in recent years. It is the only method that enables analysis of the vibrations of macromolecules which correspond to large-scale structural movements, as well as the collective dynamics (vibrational and relaxation) of hydrogen-bonded water molecules, making it indispensable in the study of aqueous solutions [17,18,19,20,21,22,23,24,25,26]. It was used to demonstrate the properties of water molecules forming a coat around biological molecules [22,27,28], which may play a role in regulating their activity [29,30,31]. The ability of water exposed to various physical factors to emit THz radiation has been revealed [8,9]. The recently demonstrated contactless effects of extremely diluted solutions of substances on biological molecules [16], and the role of THz emission in regulating biomolecule activity [32,33] were prerequisites for studying the distant effect of extremely diluted solutions on the THz characteristics of an aqueous protein solution.

Taking the above into account, we decided to evaluate the influence of extremely diluted solutions of antibodies to IFNγ on its target (namely, an aqueous solution of IFNγ) without direct contact using terahertz time-domain spectroscopy (THz-TDS). Since lactose is an excipient in the manufacturing of drugs based on diluted antibodies to IFNγ, the aqueous solution of IFNγ was studied when surrounded by a water-fluidised lactose mixture (the lactose was saturated with extremely diluted solutions of antibodies to IFNγ).

As an aside, it is worth mentioning that there are other spectral methods that can analyse changes occurring in the intermolecular structure of aqueous solutions, namely Fourier-transform IR spectroscopy (FTIR) [34,35,36] and Raman spectroscopy [37,38,39]. These methods also allow spectra in the far IR region to be obtained, partially covering the THz range. However, they are not able to determine complex dielectric functions such as the THz time-domain spectroscopy method. Also, their sensitivity in this range is much lower than that of THz spectroscopy, which has a dynamic range of 40–80 dB. FTIR and Raman spectroscopy can also be used to analyse the intermolecular characteristics of aqueous solutions by measuring spectra in the mid-IR region [38,40,41,42,43,44]. This region contains the frequencies of intramolecular vibrations of water, which depend on intermolecular binding. It should be noted that the sensitivity of these methods, one way or another, is inferior to that of THz spectroscopy.

## 2. Materials and Methods

### 2.1. Preparation of Samples

We used water obtained by a Milli-Q purification system (Millipore, Darmstadt, Germany); IFNγ-lyophilised powder of recombinant human IFNγ, 144 amino acids, molecular weight: 16.8 kDa, purity: 98% (Kozir, Moscow, Russia); antibodies to IFNγ-affinity purified rabbit polyclonal antibodies (IgG) to recombinant human IFNγ (144 amino acids), concentration: 2.5 mg/mL, purity: 99% (AB Biotechnology, Pentlands Science Park, Penicuik, Edinburgh UK); lactose monohydrate (SuperTab^®^ 30GR, DFE pharma, Goch, Germany).

It has previously been shown [45] that an aqueous solution of lactose saturated with extremely diluted solutions of antibodies to IFNγ had terahertz characteristics different from those of the control sample (an aqueous solution of lactose saturated with a solution without extremely diluted antibodies), therefore, in this study we used fluidised lactose saturated with extremely diluted solutions of antibodies to IFNγ.

Two types of samples were analysed:

1. A water-lactose mixture obtained from fluidised lactose saturated with extremely diluted solutions of antibodies to IFNγ (see below), hereinafter referred to as saturated lactose.
2. A water-lactose blend obtained from fluidised lactose saturated with extremely diluted solutions of water, designated as a control sample.

Lactose was saturated with antibodies to IFNγ, which had previously undergone a gradual reduction of their initial concentration (2.5 mg/mL) under specific conditions. Namely, antibodies to IFNγ were mixed with a solvent (an ethanol-water solution) at a ratio of 1:100 and underwent intensive vibration treatment to produce the first centesimal dilution (i.e., 100-fold dilution). All subsequent dilutions consisted of one part of the previous dilution and 99 parts of the solvent with intensive vibration treatment between each dilution. Thus, the final solution contained a mixture of 12, 30, and 50 centesimal dilutions of antibodies to IFNγ. A theoretical concentration reduction of the initial antibodies was at least 10^24^ times i.e., 2.5 × 10^−24^ mg/mL. Water subjected to a similar dilution process (hereinafter–control) was used as a control. All of the dilutions were prepared by OOO “NPF “MATERIA MEDICA HOLDING” in sterile glass vials with screw caps (Glastechnik Gräfenroda, Geratal, Germany). The resulting solution (0.8 kg) was sprayed in a fluidised bed unit on lactose powder (4 kg) and dried with warm air. The resulting saturated lactose powder was blended in a mixer with excipients to obtain a mass for tableting; compacting it in a tablet press produced experimental tablets for the study. The control sample containing no extremely diluted antibodies was prepared by applying identical procedure to purified water as initial substance. All samples (in tablet form) for testing were provided by OOO “NPF “MATERIA MEDICA HOLDING”. The samples were tested blindly and decoded after the experiment results had been obtained.

Before the study, a stock solution of IFNγ (1 mg/mL) in deionised water was prepared, and then split into 2 mL aliquots in 5 mL polystyrene tubes (Eppendorf, Hamburg, Germany). In total, 12 aliquots of IFNγ were prepared: six of them were used for experiments with lactose saturated with antibodies to IFNγ, and the remaining six were used for control experiments. Each of the six prepared samples was tested for statistical processing. Aliquots were stored at a temperature ranging from −18 to −22 °C for no more than a week and thawed at room temperature immediately before use in experiments.

Right before the experiment, the samples were prepared for testing as follows:

1. 7 mL of water was added to 40 experimental tablets (300 mg each) of each sample and left at room temperature (humidity 35%) for 15 min in sterile glass vials with screw caps (Glastechnik Gräfenroda, Geratal, Germany). Then the resulting paste was mixed with a spatula.
2. An aqueous solution of IFNγ (1 mg/mL) to the volume of 2 mL in a 5 mL polystyrene test tube was immersed in a vial with a water-lactose mixture. Thus, the part of the test tube filled with IFNγ was completely surrounded by a water-lactose mixture. There was no direct contact between the IFNγ aqueous solution and the water-lactose mixture–they were separated from each other by the wall of the test tube.
3. The vial (with the immersed polystyrene test tube of IFNγ inside it) was immersed in a Ministat 230 liquid thermostat (Huber, Offenburg, Germany), set at 37.0 °C. After one hour, 0.5 mL of the IFNγ solution was taken out of the tube for spectral analysis (sample 1, see Section 2.2). The rest of the sample was left incubated under the same conditions. After analysing the first sample, a second sample was taken for spectral analysis in a similar way. The total incubation time of the second sample was 1.5–2 h. After analysing the second sample, a third sample was taken which was incubated for 2.5–3 h. Thus, we analysed not only the effect of the saturated lactose and the control on the IFNγ aqueous solution but also the effect’s dependence on the incubation time.

It is known that water is able to change its functional characteristics under the influence of weak and superweak magnetic fields [4]. In order to minimise the effect of external undesirable physical influences on the studied samples, the same number of saturated lactose samples and control samples were tested on each day of the experiment; in general, each type of experimental sample was tested in 6 independent repeats. 

In addition, the same experiment was carried out, but the incubation time of the polystyrene test tube of IFNγ in water-lactose mixture was 3.5–4 h. Each sample was tested in seven independent repeats (18 spectral records were obtained from the Control sample and 21 spectra records were obtained for the Saturated lactose sample).

### 2.2. THz-TDS

In this study, we used the THz-TDS technique. It allows the simultaneous acquisition of absorption and refraction spectra of the test substance to calculate complex dielectric functions without using the Kramers–Kronig transformations. The details of this method are described, for example, in [46]. The spectra were recorded with a TPS Spectra 3000 spectrometer (Teraview, Cambridge, UK) in a wavenumber range from 10 to 110 cm^−1^ with a resolution of 4 cm^−1^. To obtain one spectrum, averaging over 2000 scans was performed. The humidity of the air in the room during the measurements was controlled and was approximately 35%.

The spectra of solutions were recorded in two identical cuvettes with different distances between the windows: 50.02 μm and 100.17 μm. The spectrum of the solution in the first cuvette was considered to be the background spectrum, and the spectrum of the same solution in the second cell was considered to be the spectrum of the sample. The spectrum of the sample differs from the background spectrum only in the availability of an additional 50.15 μm layer of the solution being studied. This approach to spectra recording ensures that all optical-spectral artefacts are avoided. The details of this procedure are described in [27]. Teflon gaskets were installed between the windows. The cuvette windows were made of z-cut quartz.

The exact distances between the windows were determined after assembling the cuvettes. For this, the transmission spectra of empty cuvettes at a range of 4000–7000 cm^−1^ (since the z-cut quartz is transparent in this range) were recorded on a Nicolet 6700 Fourier transform IR spectrometer (Thermo, Waltham, MA, USA). The spectra contained equidistant bands with the distance Δν due to the etalon effect. This effect was used to calculate the distance *l* between the windows according to the following formula:

(1) $$l\;\left(\mu m\right)=\frac{5000}{\Delta \nu \left({\mathrm{cm}}^{-1}\right)}.$$

The temperature of the samples during measurement was stabilised at 25 ± 0.5 °C by placing the cuvette in a thermostatic holder—a 4000 Series High stability temperature controller (Specac Inc., Orpington, UK).

After placing the cuvette with the sample in the cuvette compartment, the latter was closed with a lid, and a 10 min pause was made before measurement began. This was necessary to stabilise the temperature and purge the spectrometer with dry air. Purging was performed with an FT-IR Purge Gas Generator 74-5041 (Parker Hannifin Corporation, Haverhill, MA, USA).

### 2.3. Spectral Data Analysis

The complex function of permittivity $\varepsilon^{*}\left(\nu \right)$ was calculated from the recorded transmission spectra Tr (ν) and the refractive index n (ν), using the following formula:

(2) $$\varepsilon^{*}\left(\nu \right)=n^{2}\left(\nu \right)-{\left[\frac{\ln\mathrm{Tr}\left(\nu \right)}{4\pi \nu \Delta l}\right]}^{2}-i\times \frac{n\left(\nu \right)\ln\mathrm{Tr}\left(\nu \right)}{2\pi \nu \Delta l},\;$$

where ν is the wavenumber; i is the imaginary unit; and Δl is the difference in thickness between the windows in the two used cuvettes (50.15 μm).

In this work, we analysed the dielectric characteristics of water in IFNγ solutions (under the influence of the environment of the test samples). The dielectric permittivity $\varepsilon_{sol}^{*}$, of solutions, calculated by Formula (2), contains information about both the dielectric permittivity of water $\varepsilon_{w}^{*}$, and the dielectric permittivity of the protein $\varepsilon_{p}^{*}$ available in the solution. In this case, contributions of both components to dielectric permittivity are not additive due to mutual polarisation effects. Models of an effective medium are used to describe the dielectric properties of two-phase systems. For a continuous medium (water) with small inclusions (protein molecules), which are much smaller in size than the wavelengths of the frequency range under consideration and are close to spherical symmetry, the Bruggeman model is suitable [47]:

(3) $$f_{p}\frac{\varepsilon_{p}^{*}-\varepsilon_{sol}^{*}}{\varepsilon_{p}^{*}+2\varepsilon_{sol}^{*}}+\left(1-f_{p}\right)\frac{\varepsilon_{w}^{*}-\varepsilon_{sol}^{*}}{\varepsilon_{w}^{*}+2\varepsilon_{sol}^{*}}=0.$$

To calculate the dielectric function of the aqueous phase $\varepsilon_{w}^{*}$ from Formula (3), it is necessary to know the volume fraction of the protein *f_p_* and its complex permittivity $\varepsilon_{p}^{*}$. *f_p_* = 7.14 × 10^−4^ was calculated from the mass fraction of IFNγ (0.001) and its density. The protein density was estimated at 1.40 g/cm^3^ on the basis of the literature data [48].

From the data in [49], it is possible to extract approximate values of the real and imaginary parts of dielectric permittivity of the protein $\varepsilon_{p}^{\prime }$ and $\varepsilon_{p}^{\prime \prime }$, using the example of BSA: at 1 THz, they are 2.89 and 0.26, respectively. For water, these values at the frequency of 1 THz are about 4.6 and 2.6, and the ratios between the dielectric functions of water and protein (close to these values) remain in the frequency range from 10 to 100 cm^−1^. Thus, the value $\varepsilon_{p}^{\prime \prime }$ turns out to be an order of magnitude less than the values $\varepsilon_{w}^{\prime }$, $\varepsilon_{w}^{\prime \prime }$ and $\varepsilon_{p}^{\prime }$, included in the Formula (3), in the entire analysed frequency range. Therefore, it can be omitted to simplify calculations. We assumed that the dielectric function of the protein $\varepsilon_{p}^{*}$ contains only the real part $\varepsilon_{p}^{\prime }$. Since this value varies slightly within the range of 10 to 110 cm^−1^, we assumed it to be constant and equal to 2.89.

The solution of Equation (3) containing complex values can be obtained by dividing it into two equations according to the principle of separating the real part and the imaginary part. Thus, solving a system of two equations with real values is required. The solution to Equation (3) with complex functions was obtained in [22] and is as follows:

(4) $$\left\{\begin{array}{c} \varepsilon_{w}^{\prime }=\frac{b}{a}*\frac{c*b-a*d}{b^{2}+a^{2}}-\frac{c}{a},\\ \varepsilon_{w}^{\prime \prime }=\frac{c*b-a*d}{b^{2}+a^{2}},\\ a=\varepsilon_{p}^{\prime }+\left(2-3f_{p}\right)\times \varepsilon_{sol}^{\prime },\\ b=\left(2-3f_{p}\right)\times \varepsilon_{sol}^{\prime \prime },\\ c=\left(3f_{p}-1\right)\times \varepsilon_{p}^{\prime }\times \varepsilon_{sol}^{\prime }+2\left(\varepsilon_{sol}^{\prime \prime 2}-\varepsilon_{sol}^{\prime 2}\right),\\ d=\left(3f_{p}-1\right)\times \varepsilon_{p}^{\prime }\times \varepsilon_{sol}^{\prime \prime }-4\varepsilon_{sol}^{\prime }\varepsilon_{sol}^{\prime \prime }. \end{array}\right.$$

where $\varepsilon_{p}^{\prime },\;\varepsilon_{p}^{\prime \prime }$—the real and imaginary parts of $\varepsilon_{p}^{*}$, $\varepsilon_{sol}^{\prime },\;\varepsilon_{sol}^{\prime \prime }$—the real and imaginary parts of $\varepsilon_{sol}^{*}$. The Formulas (4) were used to calculate $\varepsilon_{w}^{\prime }$ and $\varepsilon_{w}^{\prime \prime }$—the real and imaginary parts of $\varepsilon_{w}^{*}$. Next, the dielectric constant parameters were calculated on the basis of fitting the model dielectric constant to the experimental one. The model dielectric constant was set as follows:

(5) $$\varepsilon_{w}^{*}=\frac{\Delta \varepsilon_{1}}{1-i2\pi \nu \tau_{1}}+\frac{\Delta \varepsilon_{2}}{1-i2\pi \nu \tau_{2}}+\frac{A}{\omega^{2}-{\left(2\pi \nu \right)}^{2}-i2\pi \nu \gamma }+\varepsilon_{\infty }+i\frac{\sigma_{0}}{\varepsilon_{0}2\pi \nu },$$

where $\tau_{1}$, $\Delta \varepsilon_{1}$ are the time and amplitude of Debye relaxation [50,51] and $\tau_{2}$ and $\Delta \varepsilon_{2}$ are the time and amplitude of the relaxation process related to free or weakly bound water molecules [23,52,53]; *A, ω, γ* are the amplitude, resonance frequency and damping parameter of intermolecular vibrations of water molecules bound by hydrogen bonds, respectively [54,55]; $\varepsilon_{\infty }$ is the high-frequency dielectric constant (in the region of higher frequencies relative to the vibrational band); *i* is the imaginary unit; $\sigma_{0}$ is dc-conductivity; $\varepsilon_{0}$ is the vacuum dielectric constant; and *ν* is frequency.

Some parameters of the model function (5) can be determined without using the fitting. The conductivity parameter $\sigma_{0}$ due to the unavailability of salts in the solution can be set equal to zero. The parameter of the high-frequency dielectric constant $\varepsilon_{\infty }$ can be considered constant, as it hardly changes among slightly different solutions. Moreover, its small variations do not significantly change the dielectric constant. This parameter was equated to a value of 2.5, which corresponds to the value of the real part of the water dielectric constant at a frequency of about 300 cm^−1^.

The first term in (5) describes a hydration-sensitive Debye relaxation process. When water molecules are bound into hydration shells, the amplitude decreases and the absorption maximum of this band shifts into the low frequency region. Since we are analysing a portion of the spectrum (from 10 to 110 cm^−1^) far from the maximum of the Debye relaxation band (0.6 cm^−1^) and only registering its high-frequency edge, a decrease in the amplitude $\Delta \varepsilon_{1}$ in our spectra is recorded similarly to a shift of the maximum towards lower frequencies (an increase of relaxation times $\tau_{1}$). In this regard, there is no need to consider parameters $\Delta \varepsilon_{1}$ and $\tau_{1}$ as independent and search for them both. In our calculations, we calculated the parameter $\Delta \varepsilon_{1}$, and the parameter $\tau_{1}$ was fixed at 8.28 ps, which is characteristic of water at 25 °C [56]. The remaining six parameters ($\Delta \varepsilon_{1}$, $\Delta \varepsilon_{2}$, $\tau_{2}$, A, ω, γ) were calculated using the fitting. The criterion for the fitting was minimisation of the χ value:

(6) $$\chi =\frac{1}{N}\sqrt{\sum_{i=1}^{N}\left[{\left(\frac{{\varepsilon^{\prime \prime }}^{\mathrm{ex}}\left(\nu_{i}\right)-{\varepsilon^{\prime \prime }}^{\mathrm{mod}}\left(\nu_{i}\right)}{{\varepsilon^{\prime \prime }}^{\mathrm{mod}}\left(\nu_{i}\right)}\right)}^{2}+{\left(\frac{{\varepsilon^{\prime }}^{\mathrm{ex}}\left(\nu_{i}\right)-{\varepsilon^{\prime }}^{\mathrm{mod}}\left(\nu_{i}\right)}{{\varepsilon^{\prime }}^{\mathrm{mod}}\left(\nu_{i}\right)}\right)}^{2}\right]},$$

where the indices «ex» and «mod» refer to the experimental and model dielectric functions, $\nu_{i}$—discrete wave numbers of points in the measured spectrum, *N* = 250—the number of analysed points in the spectrum.

### 2.4. Statistical Data Analysis

The distribution normality was assessed by the Shapiro-Wilk test, and homogeneity of dispersions was assessed by the Bartlett test. The samples studied were compared with the use of analysis of variance (ANOVA). The differences were considered statistically significant at *p* < 0.05.

## 3. Results

### 3.1. Dielectric Permittivities of Aqueous Phase of Analyzed IFNγ Solutions

**Figure 1:**
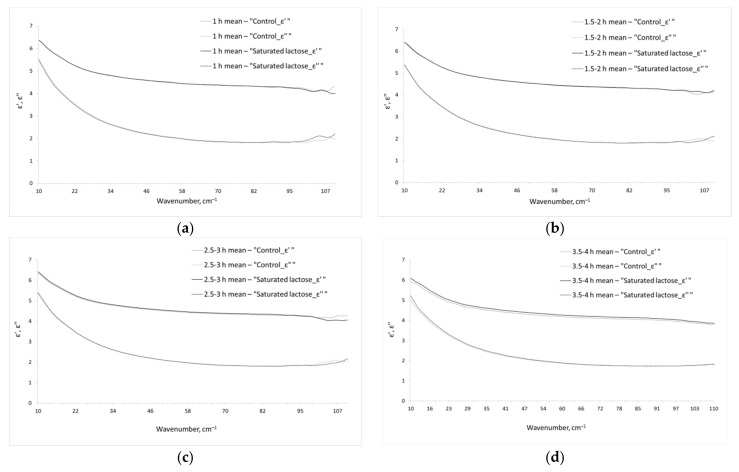
Dielectric permittivities (real ε′ and imaginary ε″ parts) of the aqueous phase of the studied IFNγ solutions. The total incubation times of IFNγ solutions in the saturated lactose (black line (ε′) or dashes (ε″)) and control (gray (ε′) or dashes (ε″)) environment are 1 h (**a**), 1.5–2 h (**b**), 2.5–3 h (**c**), 3.5–4 h (**d**).

Figure 1 shows the real and imaginary parts of dielectric permittivity of the aqueous phase of the studied IFNγ solutions. We should note that the given dielectric permittivities are related only to water phase of the solutions, since the contribution of dissolved IFNγ was excluded by effective medium model (see Section 2.3, Equations (3) and (4)). 

### 3.2. Parameters of the Model Dielectric Permittivity of the Aqueous Phase of IFNγ Solutions

As a rule, the THz spectra of solutions do not include sharp spectral bands (Figure 1), as it can be seen in mid IR or GHz ranges. This is the reason for complexity and sometimes ambiguity of the interpretation of THz spectra of the liquids. To obtain data that could be clearly interpreted, the parameters of the model dielectric permittivity (Equation (5)) were calculated, which are presented in Table 1.

Parameters of Table 1 contain the integral characteristics of dielectric permittivities and have a certain physical meaning. The analysis of these parameters of solutions compared to each other allows interpreting the intermolecular structure and dynamics of water.

Incubation of IFNγ solutions in a saturated lactose and control environment for less than 2 h did not lead to a significant difference in the calculated parameters of the model (5). However, when incubated for 2.5–3 and 3.5–4 h, statistically significant differences were found (Table 1).

Thus, statistically significant differences between the IFNγ solutions incubated for more than 2.5 h without direct contact with the saturated lactose and the control in the parameters A, ω, γ, τ_2_ were obtained.

## 4. Discussion

The findings indicate that as a result of immersing a vial with an IFNγ solution in saturated lactose for 2.5–4 h, significant structural and dynamic changes occur in the IFNγ aqueous solutions. However, no statistically significant changes were found with exposure times of less than 2 h. In order to explain the effect, it can be assumed that incubation of an intact protein solution for 2.5–4 h at 37 °C leads to conformational changes in the protein and they mediate changes in the structure of water [22]. However, the design of our experiment was such that the properties of IFNγ surrounded by the saturated lactose were compared with those of IFNγ surrounded by the control sample. In both cases, the incubation time of IFNγ and the incubation temperature were identical. In an experiment designed in such a way, it can be concluded that it is the extremely diluted solutions of antibodies to IFNγ that affect the IFNγ solution, rather than the temperature and time of incubation. 

Another possible interpretation may be the distant effect of extremely diluted solutions of antibodies to IFNγ on the molecular structure and dynamics of the water in the IFNγ solution. The contactless effect of extremely diluted solutions of antibodies to IFNγ on the IFNγ aqueous solution resulted in an increase in four parameters of the water dielectric constant (listed in Table 1). An increase in intermolecular vibration frequency ω indicates that, on average, the strength of the hydrogen bonds between water molecules has increased. An increase in parameter γ indicates an increase in the width of the distribution of hydrogen bond energies. This means that an increase in ω is associated not with a vibrational band shift, but with its expansion towards higher frequencies. Thus, the changes that occurred in the IFNγ solution entail stronger hydrogen bonds appearing between water molecules, while the weak ones remain unchanged. 

The findings agree with the results of [45], where it was also shown that the spectrum of intermolecular binding energies expands in the presence of extremely diluted solutions of antibodies to IFNγ. However, there is a significant difference between our data and the data of the aforementioned work; the effect of extremely diluted solutions of antibodies to IFNγ on water occurs not due to a direct molecular interaction, but distantly.

In addition to the parameters ω and γ, we also registered an increase in parameter A in the IFNγ solution under the influence of the emission of extremely diluted IFNγ antibody solutions. Note that from the point of view of its dielectric properties, the basic parameter is not A, but A/ω^2^. It is this relationship that determines the vibration process’s contribution to the overall dielectric response of the system, by analogy with amplitude Δε for relaxation processes. When comparing the A/ω^2^ parameter for extremely diluted solutions of antibodies to IFNγ and the reference sample, no significant differences were found. This means that the total dielectric response caused by intermolecular vibrations has not changed, rather redistribution towards higher frequencies has occurred. It follows that this change can be registered neither at a lower nor a higher frequency but only in the THz region. This once again emphasises the specificity of THz spectroscopy in studying intermolecular dynamics in aqueous solutions. 

In addition to the vibrational band parameters, we also found an increase in the relaxation time τ_2_. This relaxation process is due to dielectric relaxation of free [21] or weakly bound water molecules [57]. An increase in τ_2_ indicates an increase in the resistance of surrounding hydrogen-bonded water molecules to rotation of the free/weakly bound molecules. At the same time, there were no differences in the parameter Δε_1_. This means that the increase in hydrogen bonding recorded from the change in the intermolecular vibrational dynamics of water has practically no effect on the Debye relaxation of bound water molecules. At the molecular level, Debye relaxation consists in the simultaneous breaking of all hydrogen bonds of a water molecule with its subsequent reorientation [50,58]. At 25 °C there are 3.6 hydrogen bonds per water molecule on average [59]. That is, Debye relaxation is a rather high-energy process; therefore, small changes in the average energy of intermolecular binding cannot significantly affect it. At the same time, for free or weakly bound water molecules (with one distorted hydrogen bond), even small changes can have a noticeable effect and we can see it from the change in the parameter τ_2_.Therefore, in aqueous solution of IFNγ exposed to extremely diluted solutions of antibodies to IFNγ, more strongly bound water molecules appear. It affects free and weakly bound water molecules, leading to a slowdown in their rotational relaxation.

Thus, this paper demonstrates that dilutions of antibodies to IFNγ are able to distantly alter the structure of an IFNγ aqueous solution. The contactless effect of radiation of some systems on others has been known for a long time. Research in this field is ongoing, both in the field of biology [60,61] and in physical chemistry [16,62,63]. Presently, it would be logical to consider two possible mechanisms underlying the effect of contactless impact. The first mechanism is VRET (vibrational resonant energy transfer), which is carried out by intra- and intermolecular exchange, including through-space interaction [64]. However, VRET is very short-range, making it difficult to seriously consider it as a possible mechanism of the effect of one system on another through vessel walls. Another possible mechanism is mediated by the emission of waves. This mechanism is consistent with the results of our previous research, which showed the presence of radiation in extremely diluted solutions of antibodies to IFNγ in the IR region [16], and has also been attested by other research groups [65,66]. Although the mid-IR range carries information about intramolecular structure, it depends, in part, on intermolecular binding. This is the basis of the approach to determine the connectivity of water molecules via intramolecular vibrations [67,68]. There is now no doubt that a change in the conformation of a protein is accompanied by a change in its water shell [69], and this is inextricably linked to protein functioning [70]. The reverse is also true: a change in the water shells leads to a change in the conformation of proteins [15]. The physical mechanism of how extremely diluted solutions of antibodies to IFNγ can affect the hydration shell of proteins, causing changes in their conformation, has already been shown [15]. Taking into account the experimental data of this work, it can be concluded that the distant effect of IFNγ antibody dilutions on the properties of aqueous solutions of IFNγ leads to changes in the properties of both water and protein conformation, and, consequently, protein functions.

In addition, it has been shown that the exposure of water or aqueous-alcoholic solutions of various concentrations to a laser leads to emission in the THz region [8,71], that is, a physical effect on a liquid leads to specific radiation of the liquid. When extremely diluted solutions are obtained, a certain physical effect (vibrational action in the presence of the initial substance) is also exerted on the initial and subsequent solutions, which leads to a change in their physical-chemical properties [2,72,73,74,75], and, consequently, the solutions in general. The use of such physical effects may cause extremely high dilutions to have their own radiation with specific characteristics [16]. Thus, extremely diluted solutions having their own specific emission (depending on the initial solution being diluted) can distantly influence their target when exciting them (intensive vibration) leads to emission, and this can explain how extremely diluted solutions manifest biological activity.

More data and continued research in this field are required. Based on the findings, a longer study of the process of high dilutions’ contactless action on aqueous solutions of interferon gamma is of interest in order to determine the kinetics of the effect: the exposure dependence time. Such experiments will be the next stage of our work in this field, along with the study of other issues. The answers these questions will need to be clarified in order to understand the physics of contactless action processes.

## 5. Conclusions

This work demonstrates the possibility of the distant effect of extremely diluted solutions of antibodies to IFNγ on aqueous solutions of IFNγ. The THz-TDS technique detected changes in the structure of the aqueous solution of IFNγ during its incubation for 2.5–4 h with the specified sample without direct contact. The essence of the changes comes down to the appearance of stronger hydrogen bonds between water molecules as well as a slowdown in the dynamics of free and weakly bound water molecules. In view of the absence of a direct contact between extremely diluted solutions of antibodies to IFNγ and an IFNγ solution, an assumption was made that such an impact could be due to the emission of waves in a specific region. In continuation of this experiment, it would be appropriate to study in detail the characteristics of the experimental sample’s emission which hypothetically determines the described effect. When there is a deep understanding of the underlying mechanisms of the effect, the THz-TDS methodology could be used for quality control of pharmaceuticals based on extremely diluted solutions of antibodies.

## Figures and Tables

**Table 1:** Results of terahertz time-domain spectroscopy data analysis: model calculated parameter values (5). The data are presented as mean ± SD.

Incubation Time	Sample	Δε_1_	Δε_2_	τ_2_*10, ps	A/10^3^, cm^−2^	A/ω^2^	ω, cm^−1^	γ, cm^−1^
1 h	Control	71.03 ± 4.08	2.63 ± 0.13	3.06 ± 0.28	72.00 ± 20.62	1.68 ± 0.15	204.50 ± 21.69	190.00 ± 54.59
	Saturated lactose	67.52 ± 4.49	2.76 ± 0.07	3.12 ± 0.16	57.00 ± 13.67	1.64 ± 0.11	185.33 ± 15.88	154.17 ± 37.34
1.5–2 h	Control	67.08 ± 3.97	2.74 ± 0.15	3.13 ± 0.15	70.67 ± 21.61	1.68 ± 0.16	202.33 ± 22.57	189.17 ± 46.20
	Saturated lactose	66.18 ± 2.70	2.77 ± 0.15	3.19 ± 0.25	70.67 ± 11.15	1.70 ± 0.07	203.00 ± 11.85	188.33 ± 29.10
2.5–3 h	Control	67.30 ± 5.97	2.68 ± 0.12	2.92 ± 0.16	50.67 ± 7.23	1.58 ± 0.08	178.67 ± 9.18	134.17 ± 19.85
	Saturated lactose	66.65 ± 2.43	2.79 ± 0.15	3.22 ± 0.15 * (*p* = 0.007)	67.33 ± 11.57 * (*p* = 0.01)	1.70 ± 0.10	198.33 ± 12.99 * (*p* = 0.01)	180.00 ± 28.64 * (*p* = 0.009)
3.5–4 h	Control	67.91 ± 8.60	2.34 ± 0.22	2.74 ± 0.21	53.84 ± 22.82	1.46 ± 0.36	188.17 ± 16.87	174.57 ± 32.99
	Saturated lactose	67.24 ± 9.26	2.49 ± 0.22 * (*p* = 0.042)	3.03 ± 0.28 * (*p* < 0.001)	74.10 ± 23.93 * (*p* = 0.035)	1.57 ± 0.21	212.94 ± 26.93 * (*p* = 0.004)	222.34 ± 46.03 * (*p* < 0.001)

* Statistically significant difference from the corresponding control at the corresponding time (the corresponding *p*-value is indicated in parentheses).

## Data Availability

The data presented in this study are available on request from the corresponding author.
